# Molecular Segmentation of the Spinal Trigeminal Nucleus in the Adult Mouse Brain

**DOI:** 10.3389/fnana.2021.785840

**Published:** 2021-12-10

**Authors:** Isabel M. García-Guillén, Margaret Martínez-de-la-Torre, Luis Puelles, Pilar Aroca, Faustino Marín

**Affiliations:** Department of Human Anatomy and Psychobiology, Faculty of Medicine, Regional Campus of International Excellence “Campus Mare Nostrum”, Biomedical Research Institute of Murcia (IMIB-Arrixaca), University of Murcia, Murcia, Spain

**Keywords:** hindbrain, somatosensory system, trigeminal system, rhombomeres, transcription factors, tachykinins, calcium-binding proteins, fibronectin

## Abstract

The trigeminal column is a hindbrain structure formed by second order sensory neurons that receive afferences from trigeminal primary (ganglionic) nerve fibers. Classical studies subdivide it into the principal sensory trigeminal nucleus located next to the pontine nerve root, and the spinal trigeminal nucleus which in turn consists of oral, interpolar and caudal subnuclei. On the other hand, according to the prosomeric model, this column would be subdivided into segmental units derived from respective rhombomeres. Experimental studies have mapped the principal sensory trigeminal nucleus to pontine rhombomeres (r) r2-r3 in the mouse. The spinal trigeminal nucleus emerges as a plurisegmental formation covering several rhombomeres (r4 to r11 in mice) across pontine, retropontine and medullary hindbrain regions. In the present work we reexamined the issue of rhombomeric vs. classical subdivisions of this column. To this end, we analyzed its subdivisions in an AZIN2-lacZ transgenic mouse, known as a reference model for hindbrain topography, together with transgenic reporter lines for trigeminal fibers. We screened as well for genes differentially expressed along the axial dimension of this structure in the adult and juvenile mouse brain. This analysis yielded genes from multiple functional families that display transverse domains fitting the mentioned rhombomeric map. The spinal trigeminal nucleus thus represents a plurisegmental structure with a series of distinct neuromeric units having unique combinatorial molecular profiles.

## Introduction

The morphological and functional complexity of the vertebrate brain first arises from early neural plate or tube stages, when the primordia for the different transverse and longitudinal regions and subregions start their specification and differentiation. One of the mechanisms involved in the increasing complexity of the brain anlagen is the segmentation process, that is, the subdivision of tagmata and proneuromeres (Puelles et al., 2018) into transverse domains (neuromeres) along the rostrocaudal axis. These brain segments or neuromeres display different molecular and cellular identities, and develop via proliferation and neurogenesis into distinct portions of the brain containing specific neuronal populations (Puelles et al., 1987, 2013; Lumsden, 1990; Kiecker and Lumsden, 2005). The diverse segmental domains are maintained through development, albeit given migratory processes may cause some violation of their boundaries, as shown by fate mapping experiments (Birgbauer and Fraser, 1994; Marín and Puelles, 1995; Watson et al., 2017). The current segmental paradigm we use for the vertebrate brain is the prosomeric model, which considers 7 prosomeres in the forebrain (including two midbrain mesomeres), and the isthmus plus 11 rhombomeres (r) in the hindbrain (Watson et al., 2010; Puelles et al., 2013, 2018; Nieuwenhuys and Puelles, 2016; Ten Donkelaar, 2020).

The modern segmental conception of the brain leads to the explanation of any columnar structures longer than a segment as plurisegmental complexes subdivided into either manifest or hidden segmental subunits or modules that would each derive from a specific neuromere. One of such columnar structures is represented in the alar hindbrain by the trigeminal sensory complex or trigeminal column. This receives and analyzes the primary somatosensory afferents from the trigeminal ganglion. This column is formed by the principal trigeminal sensory nucleus (Pr5), located in a rostral portion of the hindbrain (in the pontine region), and the spinal trigeminal sensory nucleus (Sp5), which extends caudalwards down to the hindbrain/spinal cord boundary. The Sp5 is subdivided conventionally into oral, interpolar and caudal subnuclei (pars oralis, interpolaris and caudalis of Olszewski, 1950; Sp5O, Sp5I, Sp5C), which were first defined according to cytoarchitectural characteristics (Olszewski, 1950), and display distinctive molecular markers and connectivity patterns (Waite, 2004). The rostrocaudal organization of successive Pr5, Sp5O, Sp5I, and Sp5C units raises the question of the possible correspondence of their mutual boundaries with interrhombomeric limits. Moreover, the hypothesis may be considered that the underlying rhombomeric scaffold may actually establish a finer, segment-by-segment organization of the trigeminal column.

Previous approaches have discerned part of the segmental (rhombomeric) organization of this nuclear complex. In the chick, fate mapping results (Marín and Puelles, 1995; Aroca et al., 2006) and gene expression analysis together with axon labeling data (Rhinn et al., 2013) show that Pr5 is located in r1. However, in mice Pr5 appears within r2 and r3, as concluded from genetic lineage tracings (Oury et al., 2006). Therefore, it can be deduced that Sp5, which limits rostrally with Pr5, covers either from r2 in the chick or r4 in the mouse, down to r11. Fate mappings in avian chimeras showed that Sp5 is formed indeed by successive rhombomeric units (Marín and Puelles, 1995; Cambronero and Puelles, 2000). These units display a graded differential expression of Hox genes, coherently with the respective rhombomeric identities, as described at late gestational stages in chick and mouse (Marín et al., 2008; Tomás-Roca et al., 2016). Therefore, this rhombomeric pattern is documented at least in a differential Hox code for these units of the trigeminal column. Considering the crucial role of Hox genes, as well as other gene families related to hindbrain segmentation (Addison and Wilkinson, 2016; Parker and Krumlauf, 2020), it is possible that the segmental units of the trigeminal column develop characteristic molecular and cellular identities, with probable neurochemical and/or functional implications.

In the present work we explored the possibility of dividing the trigeminal column into multiple segmental, transverse domains according to molecular criteria. To this end, we searched the Allen Mouse Brain Atlas (AMBA) (Lein et al., 2007) and the Allen Developing Mouse Brain Atlas (ADMBA) (Thompson et al., 2014) for genes with a segmental pattern within this structure. The molecular domains we found were analyzed in relation to the known neuromorphological markers of rhombomeres according to previous work of our lab cited above. We delimited as well the trigeminal segmental map in the brain of the AZIN2-lacZ transgenic mouse, which was already used previously as a working model for hindbrain cyto- and genoarchitecture (Martínez-de-la-Torre et al., 2018), and examined in parasagittal sections the pattern of labeled trigeminal afferent fibers in transgenic reporter lines retrieved from the Gene Expression Nervous System Atlas (GENSAT) (Gong et al., 2003). On the whole we propose a novel segmental map of the mouse trigeminal column according to gene expression, characterizing it as a plurineuromeric modular complex in relation to a series of rhombomere-derived domains.

## Materials and Methods

### Transgenic Mice

All experimental protocols and handling, use, and care of laboratory animals were conducted in compliance with the current normative standards of the European Union (Directive 2010/63/EU), the Spanish Government (Royal Decree 1201/2005 and 53/2013; Law 32/107) and had the approval of the University of Murcia Committee for Animal Experimental Ethics.

We used adult brains of a heterozygotic mice line developed at the Department of Biochemistry, School of Medicine, University of Murcia (López-Garcia et al., 2013). These mice express recombinant beta-galactosidase protein under control of the *Azin2* promoter. After standard perfusion, dissection and embedding in agarose, vibratome 120 μm thick serial sections were obtained. Serial sections were obtained in either sagittal or horizontal planes. Floating sections were then reacted for beta-galactosidase and were finally washed, dehydrated, mounted on slides, and covered.

Digital microphotographs were acquired using Aperio CS2 technology (Leica Microsystems GmbH, Mannheim, Germany).

### Mining of the Gene Expression Nervous System Atlas Database

This database provides images from transgenic mice lines with EGFP as reporter for the expression of diverse genes. According to the recorded GENSAT procedures, the brain sections were processed for immunohistochemistry against EGFP, which normally leads to full labeling of the positive neurons including their soma and fibers. We searched for genes expressed in the trigeminal ganglion, whose axons reach the brain and form the ascending and descending trigeminal tracts, delimiting in this way the extent of the trigeminal sensory column. We selected two specimens, corresponding, respectively, to *Calca* and *Avil* reporter lines. We downloaded the selected images, and cropped them to show our region of interest.

### Mining of the Allen Brain Database

We searched the AMBA and ADMBA for genes whose respective *in situ* hybridization (ISH) experiments included both sagittal and coronal section series at P56^1^ as well as sagittal section series from stages P4, P14, and/or P28.^2^ We screened these image series visually, selecting the genes with significant expression within subregions of Sp5. This analysis was initially carried out by two of the authors independently, whose preliminary results were discussed to reach a consensus. Our criteria to select genes was firstly that they displayed discrete expression patterns, with positive and negative regions visible along the longitudinal axis of Sp5; secondly, we checked that these patterns were coherently reproduced in both sagittal and coronal P56 stage series, as well as in the juvenile series (P4, P14 and/or P28). For some of the selected genes, part of the image series from these juvenile stages displayed generalized low ISH signal or high background, so that these image series were discarded from analysis.

As a result, we identified 12 genes (Table 1) that were differentially expressed in the trigeminal column, whose image series were analyzed, including the brightfield microphotographs or scanned images as well as their respective counterpart with color-coding of the expression level, both of them available for each brain section. In the brightfield images, the positive cells appear with a blue precipitate accordingly to standard ISH protocols. The sections corresponding to juvenile stages (P4, P14, and P28) are counterstained with HP Yellow. In the color-coded images from adult and juvenile stages, the expression intensity ranges from blue (low expression intensity), through green (medium intensity) to red (high intensity).

**TABLE 1:** Summary of the expression of each gene in the rhombomeric portions of the trigeminal column.

Figure	Gene	r2	r3	r4	r5	r6	r7	r8	r9	r10	r11	my1
4A–C	*Baiap3*	+								+++	+++	+++
4D–G	*Camk2a*							+	++	+++	+++	+++
5	*Irx2*	++	+				+	+	+			
6	*Kcng4*	+++	+++	+	+	+	++	++	+++			
7	*Mafb*			++	+++	+ ++	++	+				
8	*Fn1*			+ +	+++	+ ++	++	+				
9A–C	*Tac1*									++	++	++
9D–F	*Tac2*									+	++	++
10A–I	*Calb1*				+	+	+	++	+++	+++	+++	+++
10J–R	*Calb2*	+++	+++	+	+	+	+	+		+++	+++	+++
11A–D	*Pde1c*								++			
11E–N	*Zbtb16*								++			

*+, ++, +++ indicate three semiquantitative relative levels of expression from low to high.*

We downloaded the images that included the whole or part of the trigeminal column, cropping them to show the region of interest. In our figures, the images from parasagittal sections are oriented with the rostral end to the left, while those from coronal sections are details of the right side of the original image, with the midline to the left.

The list of the selected genes, together with the references of their respective experiments and downloaded images from the AMBA and ADMBA databases, is indicated in Supplementary Table 1.

## Results

**FIGURE 1:**
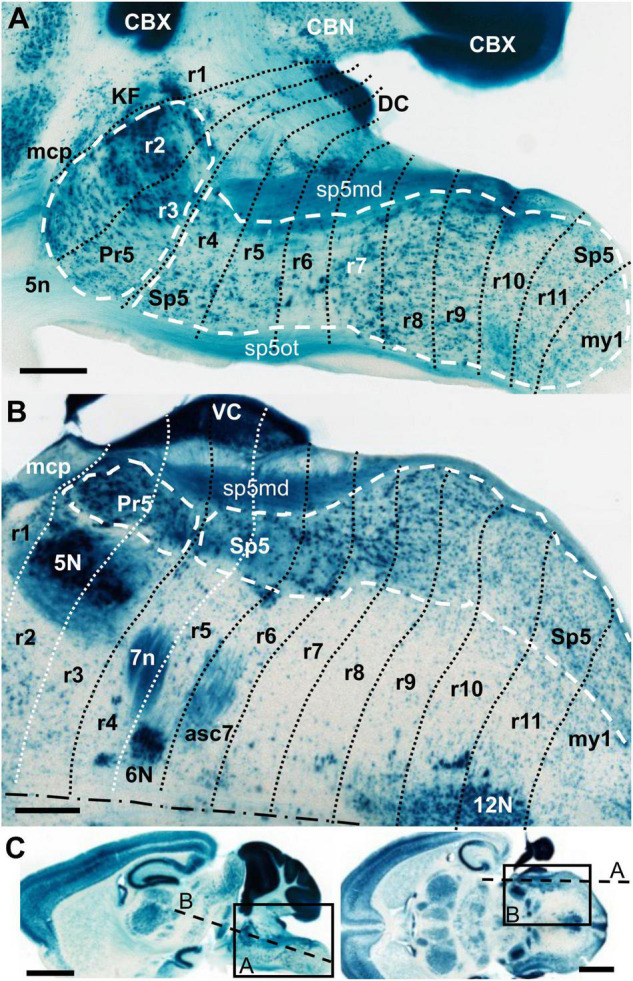
AZIN2-LacZ expression in the adult hindbrain. **(A,B)** Respective details of parasagittal and horizontal sections. In each of them the rostral end is to the left. The black or white dotted lines indicate interrhombomeric boundaries. The white dashed lines delimit the principal sensory (Pr5) and the spinal trigeminal nucleus (Sp5). The dot-dash line in B indicates the midline. **(C)** Images of the original full sections. The rectangles delimit the respective positions of A and B images. The dashed lines indicate the respective approximate positions of their sectioning planes. 5n, trigeminal nerve root; 5N, trigeminal motor nucleus; 6N, abducens motor nucleus; 7n, facial nerve; 12N, hypoglossal motor nucleus; asc7, facial ascending fibers; CBN, cerebellar nuclei; CBX, cerebellar cortex; DC, dorsal cochlear nuclei; KF, Kölliker-Fuse nucleus; mcp, middle cerebellar peduncle; r, rhombomere(s); sp5md, mandibular fibers of the trigeminal tract; sp5ot, ophthalmic fibers of the trigeminal tract; VC, ventral cochlear nuclei. **(A,B)** Scale bars = 500 μm, **(C)** scale bars = 2,000 μm.

**FIGURE 2:**
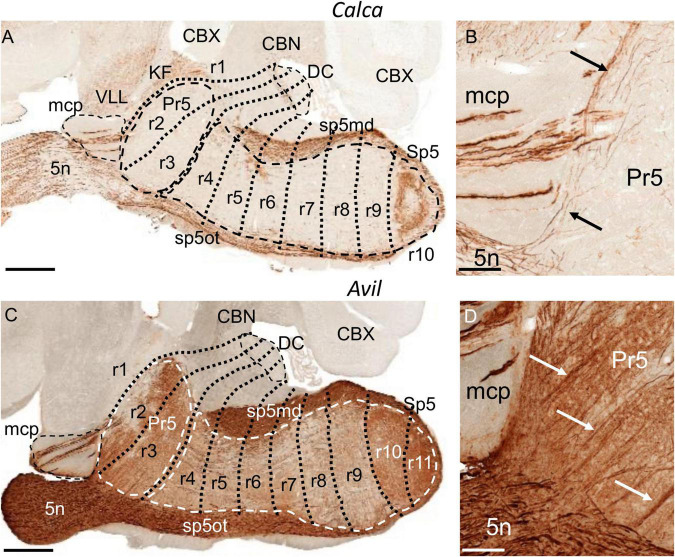
Labeling of trigeminal primary afferent fibers in adult transgenic mice from the GENSAT database, processed for immunohistochemical detection of EGFP. **(A,C)** Respective parasagittal sections of adult brains from *Calca* and *Avil* mouse lines. The principal sensory (Pr5) and the spinal trigeminal nucleus (Sp5), the dorsal cochlear nuclei (DC) and the medial cerebellar peduncle (mcp) are, respectively, encircled by dashed lines. In addition to the trigeminal system, there is expression in the ventral nucleus of the lateral lemniscus (VLL) and in the Kölliker-Fuse nucleus (KF) in the section from the *Calca* line. **(B,D)** Respective details of the former sections, showing labeling in the trigeminal nerve root (5n), some labeled fibers within mcp, and labeled ascending trigeminal fibers (black and white arrows) within Pr5. CBN, cerebellar nuclei; CBX, cerebellar cortex; r, rhombomere(s); sp5md, mandibular fibers of the trigeminal tract; sp5ot, ophthalmic fibers of the trigeminal tract. **(A,C)** Scale bars = 500 μm, **(B,D)** scale bars = 100 μm.

**FIGURE 3:**
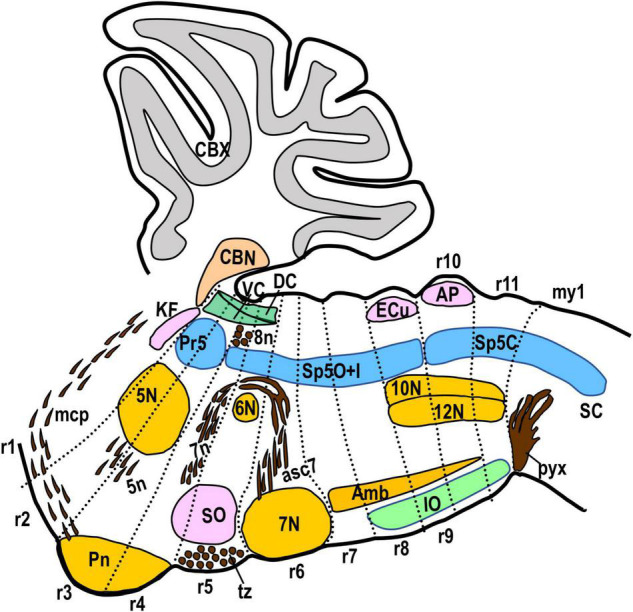
Schematic drawing showing a sagittal view of the hindbrain plus the first myelomere with the morphological landmarks used to delimit the rhombomeric domains in this work. The Kolliker-Fuse nucleus (KF), the cerebellar cortex and nuclei (CBX, CBN) and the ventral nucleus of the lateral lemniscus (VLL) are located in r1. The medial cerebellar peduncle (mcp) crosses superficially r2 and r1. The dorsal and ventral cochlear nuclei (DC, VC) extend from r2 to r5. The trigeminal motor nucleus (5N) and the root of the trigeminal nerve (5n) are located across r2 and r3. The pontine nuclei (Pn) are located superficial and medially in r3 plus r4. The bundles of fibers of the vestibulocochlear nerve (8n) and the descending fibers of the facial nerve (7n) are in r4. The abducens motor nucleus (6N) is medially in r5. The superior olive (SO) and the trapezoid body (tz) are located principally in r5. The facial motor nucleus (7N) is located in r6, with its rostral and caudal ends bulging, respectively, into r5 and r7. The inferior olive (IO) extends from r8 to r11. The complex formed by the hypoglossal and vagal motor nuclei (12N, 10N) is located in r9, r10, and r11. The ambiguous motor nucleus (Amb) extends from r7 to r10. The external cuneatus nucleus (ECu) appears in r9 at lateral section levels. The area postrema (AP) is located at the midline in r10. The pyramidal decussation (pyx) is located in the first myelomere (my1). Concerning the trigeminal column, the principal sensory nucleus (Pr5) is located in r2 and r3, while the spinal trigeminal nucleus (Sp5) extends from r4 caudalwards. Within Sp5, the limit between interpolar and caudal subnuclei is located at the r9/r10 limit. We have drawn Sp5 including the dorsal horn of the first myelomere, considering their similarity in morphology and gene expression.

**FIGURE 4:**
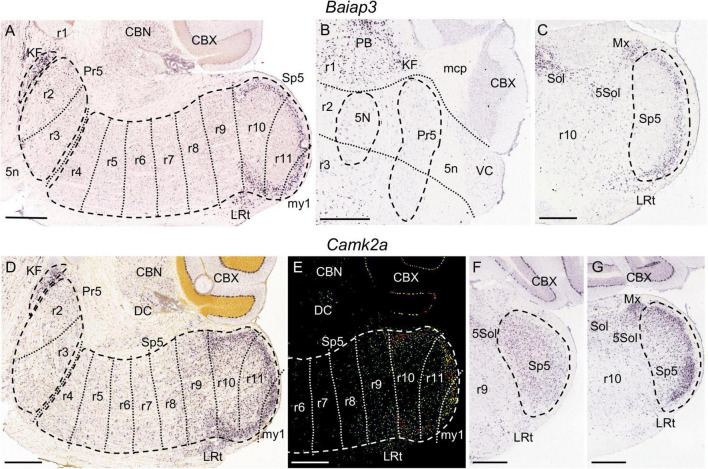
*Baiap3* **(A–C)** and *Camk2a* **(D–G)** expression. **(A)** Parasagittal section from adult brain at a lateral level showing the full extent of the trigeminal column (Pr5 plus Sp5, respectively, encircled by dashed lines). KF is also encircled by dashed lines. In this and following parasagittal sections, each of the proposed interrhombomeric boundaries (dotted lines) is represented only within the trigeminal column regarding the objective of this work. There is *Baiap3* expression in KF as well as in the r10 and r11 portions of the trigeminal column, concretely in its gelatinous layer. In **(A,D,E)** these parasagittal planes include only a small and superficial portion of my1, because this segment lies medially as compared to the rhombomeres, accordingly to the small mediolateral diameter of the spinal cord as compared to the hindbrain (see Figure 1B). **(B)** Coronal section from adult brain at the level of the trigeminal motor nucleus (5N) crossing through r1, r2 and r3. There is *Baiap3* expression in KF and other parabrachial nuclei (PB) within r1, while Pr5 (encircled by dashed lines) remains negative in this section. **(C)** Coronal section from adult brain at the level of r10, showing *Baiap3* expression in the gelatinous layer of Sp5 (encircled by dashed lines) as well as in the lateral reticular nucleus (LRt), the solitary nucleus (Sol), the trigeminal-solitary transition zone (5Sol) and the matrix region of the medulla (Mx). **(D,E)** Respective brightfield and color-coded images of a parasagittal section from a P28 brain processed for detection of *Camk2a* expression. Besides KF and LRt, there is expression in the r10 and r11 portions of Sp5, with a laminar pattern showing higher intensity in its gelatinous layer. The r9 portion of Sp5 express this gene homogeneously, with a gradient expression extending into r8. In the rest of rhombomeres, from r2 to r7, there is expression in some scattered cells. **(F,G)** Respective coronal sections from adult brain at r9 and r10 levels of an adult brain, displaying their aforementioned expression pattern in the respective portions of Sp5. Positive zones for *Camk2a* expression close to Sp5 include Sol, Sol5, Mx and LRt. Scale bars = 500 μm.

**FIGURE 5:**
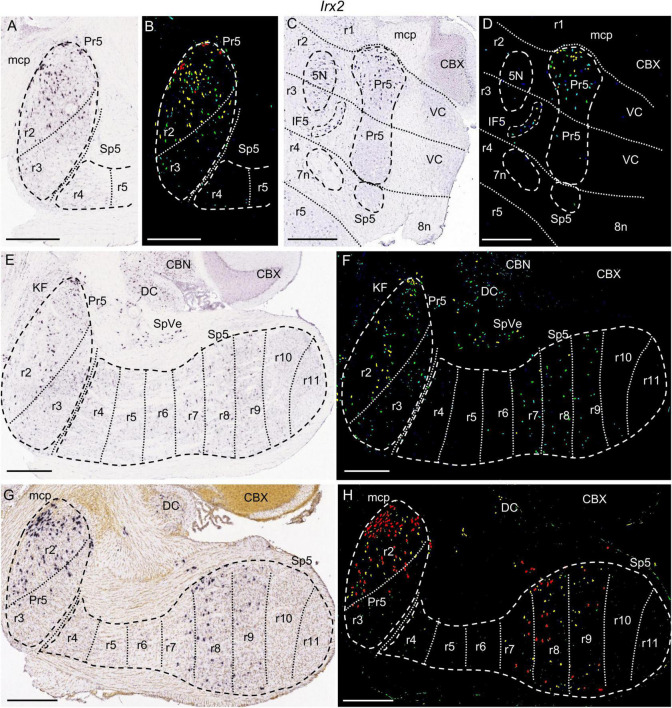
*Irx2* expression. In each image Pr5 and/or Sp5, as well as other structures indicated below, are, respectively, encircled by dashed lines. **(A–D)** Details of the expression pattern from an adult brain in Pr5, with its r2 portion expressing this gene, while r3 has only some positive cells. **(A,B)** Brightfield and color-coded images from a parasagittal section at lateral level. **(C,D)** Brightfield and color-coded images from a coronal section. R2 and r3 are delimited according to the position of 5N across both rhombomeres. 7n and 8n bundles determine the position of r4. Besides the r2 trigeminal portion, there is also expression in IF5. 5N, 7n, and IF5 are, respectively, encircled by dashed lines. **(E,F)** Brightfield and color-coded images from a parasagittal section, medial to the one shown in A. There is mild expression in the r2 and r3 trigeminal portions, with more positive cells in the former. Caudally, r7, r8, and r9 display some positive cells. **(G,H)** Brightfield and color-coded images from a parasagittal section of a P14 brain. The pattern is similar to the one shown for the adult in the former figures, but with higher intensity and a greater number of labeled cells in the positive zones (r2, r7, r8, and r9 portions of the trigeminal column). Scale bars = 500 μm.

**FIGURE 6:**
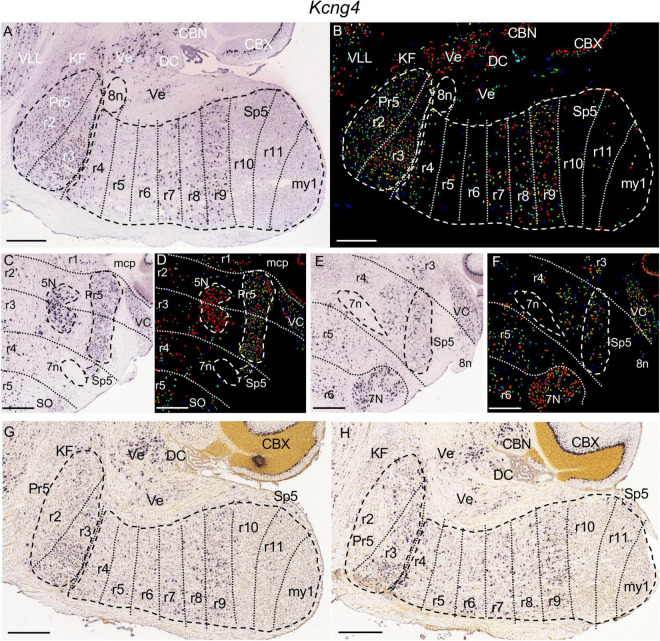
*Kcng4* expression. **(A,B)** Brightfield and color-coded images from a parasagittal section from an adult brain. Pr5 and Sp5, as well as the 8n bundle, are, respectively, encircled by dashed lines. There is homogenous expression within the r2 and r3 portions of Pr5, as well as the r9 portion of Sp5. From r9 the expression extends gradientally into the r8 and r7 portions of Sp5. There are also some disperse labeled cells within the r4, r5 and r6 portions of Sp5. Outside the trigeminal territory, in the brainstem this gene is expressed in KF, DC and vestibular nuclei (Ve). **(C,D)** Brightfield and color-coded images of a coronal section from an adult brain at the level of 5N. This nucleus, as well as Pr5, Sp5 and 7n are, respectively, encircled by dashed lines. There is dense and homogenous expression within the r2 and r3 portions of Pr5. The r4 portion of Sp5, adjacent to 7n, has some labeled cells. **(E,F)** Brightfield and color-coded images of a coronal section from an adult brain at the level of 7N. The limits of r4 are tentatively drawn enclosing the positions of the intraencephalic portion of 7n and the entry root of 8n. The r4 portion of Sp5 displays some labeled cells. This gene shows also expression in 5N and 7N as well as in VC. **(G,H)** Brightfield images of parasagittal sections, respectively, from P14 **(G)** and P28 **(H)** brains. There is *Kcng4* expression in Pr5, although in the r2 portion of this nucleus the labeled cells are located only in its dorsal region. Within Sp5 the expression pattern from r4 to r9 is similar to that described for the adult brain. Scale bars = 500 μm.

**FIGURE 7:**
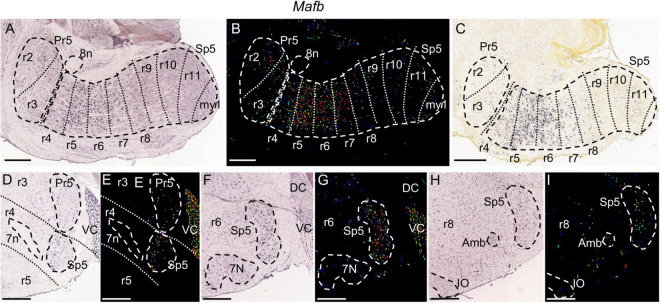
*Mafb* expression. **(A,B)** Brightfield and color-coded images of a parasagittal section of an adult brain. Pr5 and Sp5, as well as the 8n bundle, are, respectively, encircled by dashed lines. There are sparse labeled cells along all the trigeminal column, from the r2 to the r11 trigeminal portions. The expression is strongest in the r5 and r6 portions of Sp5, showing also moderate labeling in r4 and in r7 plus r8 within Sp5. **(C)** Brightfield image of a parasagittal section from a P4 brain, showing the aforementioned pattern described in the adult. **(D–I)** Pairs of brightfield and color-coded images of coronal sections from an adult brain, respectively, at the level of r4 **(D,E)**, r6 **(F,G)** and r8 **(H,I)**. The principal landmarks used for their respective identification are 7n for r4, 7N for r6 and the rostralmost end of the inferior olive (IO) plus the ambiguous motor nucleus (Amb) for r8. This gene shows also significant expression in VC **(D–G)**. Scale bars = 500 μm.

**FIGURE 8:**
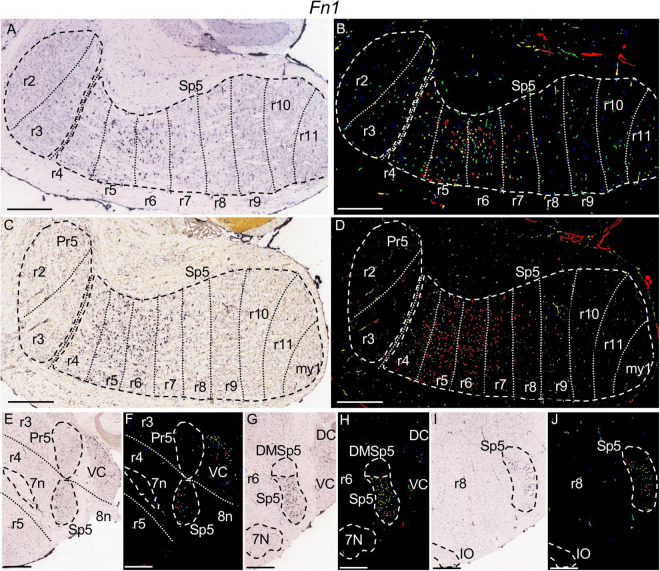
*Fn1* expression. In each image Pr5 and/or Sp5, as well as other structures indicated below, are, respectively, encircled by dashed lines. **(A–D)** Pairs of brightfield and color-coded images of parasagittal sections, respectively, from adult **(A,B)** and P28 **(C,D)** brains. The rhombomeric expression pattern within the trigeminal column is similar to that described for *Mafb* in the former figure. **(E,F)** Brightfield and color-coded images of a coronal section from an adult brain at the level of r4, identified by the positions of the fibers of the facial (7n) (encircled by dashed lines) and vestibulocochlear (8n) nerves. There is moderate labeling in Sp5 and in the ventral cochlear nuclei (VC). **(G,H)** Brightfield and color-coded images of a coronal section from an adult brain at the level of r6. 7N and Sp5, as well as the dorsomedial subdivision of the latter (DMSp5) are, respectively, encircled by dashed lines. There appears strong expression in Sp5, excluding DMSp5 which lacks expression of this gene. **(I,J)** Brightfield and color-coded images of a coronal section from an adult brain at the level of r8, showing weak expression in the portion of Sp5 in this rhombomere. Sp5 and the inferior olive (IO) are, respectively, encircled by dashed lines. Scale bars = 500 μm.

**FIGURE 9:**
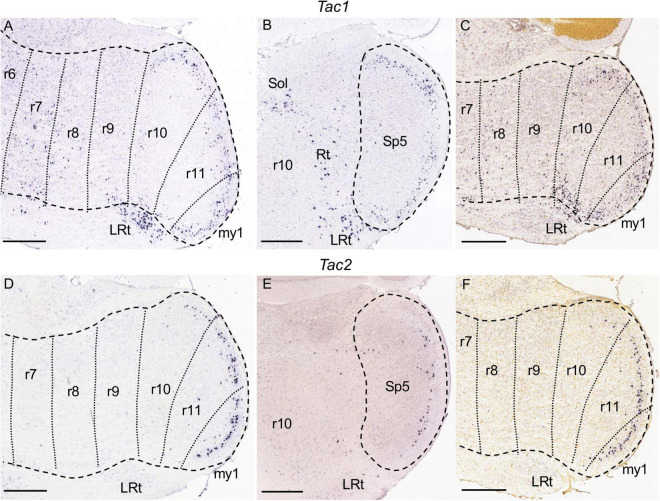
*Tac1* **(A–C)** and *Tac2* **(D–F)** expression. In each image Pr5 and/or Sp5 are encircled by dashed lines. **(A–C)** Brightfield image details of a parasagittal **(A)** and a coronal **(B)** section from adult brains, and of a parasagittal section from a P28 brain **(C)** processed for detection of *Tac1* expression. There is expression in the gelatinous layer of the r10 and r11 portions of Sp5, as well as in their caudal continuation in my1. Additionally, there are sparse positive cells from r7 to r9. There is expression also in the part of the lateral reticular nucleus (LRt) close to Sp5, as well as disperse cells in the solitary nucleus (Sol) and the medullary reticular formation (Rt). **(D–F)** Counterparts of the former images at similar levels, processed for *Tac2* detection. There is a decreasing graded expression in the gelatinous layer of Sp5 from my1 to r10, while the other rhombomeres are negative. Scale bars = 500 μm.

**FIGURE 10:**
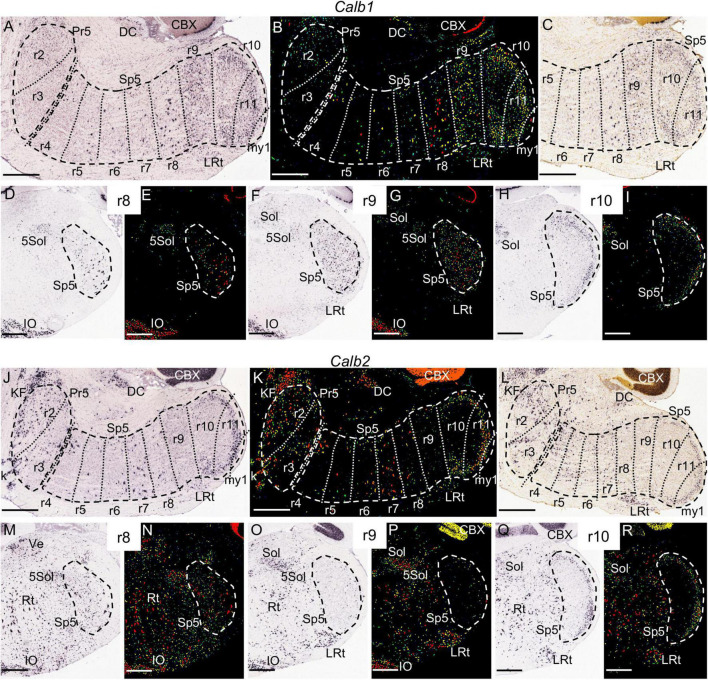
*Calb1* **(A–I)** and *Calb2* **(J–R)** expression. In each image Pr5 and/or Sp5 are encircled by dashed lines. **(A,B)** Brightfield and color-coded images of a parasagittal section of an adult brain. There is homogenous expression in the r9 portion of Sp5, as well as a laminated pattern in r10 and r11 with higher intensity in the gelatinous layer. Additionally, there are some scattered positive in the trigeminal column across the rest of rhombomeres, principally in r8. In **(A,B,J,K)** the section plane would include only a small portion of my1 as commented for Figures 4A,D,E. **(C)** Brightfield image of a P28 parasagittal section, reproducing the aforementioned *Calb1* pattern. **(D–I)** Pairs of brightfield and color-coded images of respective coronal sections of an adult brain at the levels of r8, r9 and r10. Besides the aforementioned pattern in the trigeminal column, in these section planes there is expression in the inferior olive (IO), the solitary nucleus (Sol), the trigeminal-solitary transition zone (5Sol) and part of the lateral reticular nucleus (LRt). **(J,K)** Brightfield and color-coded images of a parasagittal section of an adult brain processed for detection of *Calb2* expression. There is expression in the r10 and r11 portions of Sp5, showing a laminated pattern with higher intensity in the gelatinous layer, together with medium-high intensity in r2 and r3, and scattered positive cells from r4 to r8. **(L)** Brightfield image of a P28 parasagittal section, reproducing the aforementioned *Calb2* pattern. **(M–R)** Pairs of brightfield and color-coded images of respective coronal sections of an adult brain at the levels of r8, r9 and r10. Note the absence of *Calb2* expression in r9, as compared to scattered cells in r8 and a laminar pattern in r10. There is also expression in the inferior olive (IO), the solitary nucleus (Sol), the trigeminal-solitary transition zone (5Sol), the lateral reticular nucleus (LRt) and other parts of the medullary reticular formation (Rt). Scale bars = 500 μm.

**FIGURE 11:**
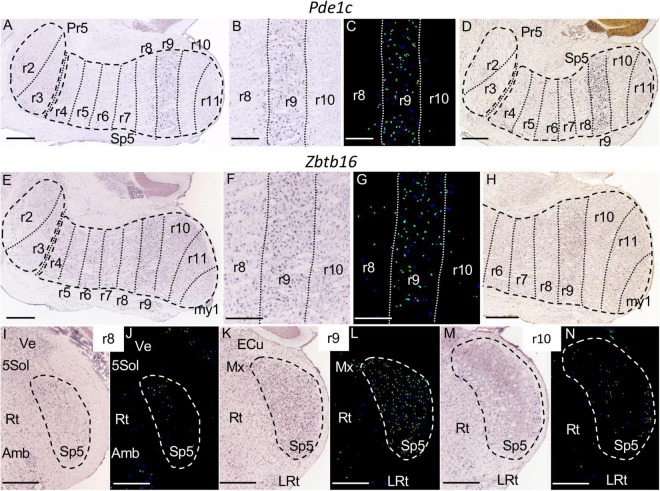
*Pde1c* **(A–D)** an *Zbtb16* **(E–N)** expression. **(A)** Brightfield image of a parasagittal section of an adult brain processed for *Pde1c* detection, showing specific expression in the r9 portion of Sp5. **(B,C)** Magnification detail of the latter image, together with its color-coded counterpart, centered in r9. **(D)** Brightfield image of a parasagittal section from a P28 brain, showing the aforementioned expression pattern for *Pde1c*. **(E)** Brightfield image of a parasagittal section of an adult brain processed for *Zbtb16* detection, showing specific expression in the r9 portion of Sp5. **(F,G)** Magnification detail of the latter image, together with its color-coded counterpart, centered in r9. **(H)** Brightfield image of a parasagittal section from a P28 brain, showing the aforementioned expression pattern for *Zbtb16*. **(I–N)** Pairs of brightfield and color-coded images of respective coronal sections of an adult brain at the levels of r8, r9, and r10. Note the specific expression of *Zbtb16* in the r9 portion of Sp5, as compared to few positive cells in the r8 and r10 portions of this nucleus. Other structures close to Sp5 -external cuneatus nucleus (ECu), Ve, 5Sol, Rt, Amb and LRt- are indicated as reference landmarks. **(A,D,E,H–N)** Scale bars = 500 μm; **(B,C,F,G)** scale bars = 300 μm.

We first analyzed the sagittal and horizontal section series of AZIN2-lacZ transgenic brains, as a basis for the delimitation of the rhombomeric map. To this end we defined the segmental domains according to the available morphological landmarks (Figure 1). Next, we proceeded to the analysis of parasagittal sections from the selected GENSAT transgenic mice, tracing the primary afferent trigeminal fibers (Figure 2). A scheme summarizing the morphological landmarks we have used appears in Figure 3. The core of the present work, our screening of AMBA and ADMBA databases, yielded 12 selected genes with regionalized, segment-related expression domains covering one or several rhombomeric domains (Figures 4–11).

We next describe the subdivisions of the trigeminal sensory complex in relation to the prepontine, pontine, retropontine and medullary proneuromeric regions of the hindbrain, with their respective component rhombomeres (Watson et al., 2019). Although the objective of this work was to propose a rhombomeric map for Sp5, we have included in our analysis also Pr5 since it represents the rostral continuation of the trigeminal column. A summary of the rhombomeric pattern of each of the analyzed genes appears in Table 1.

### Prepontine Region (Isthmus and r1) and r1/r2 Boundary

In the mouse, the alar isthmocerebellar region forms rostrally the isthmus and caudodorsally the whole cerebellum, including the cerebellar cortex (CBX) and nuclei (CBN) (Sgaier et al., 2005) (Figures 1A, 2A,C, 3). The Pr5 is entirely formed by r2 and r3 in the mouse (Figures 1A,B, 2A,C, 3), with the ascending afferent trigeminal fibers stopping at the r1/r2 boundary (Oury et al., 2006). Therefore, in the mouse Pr5 abuts directly r1 structures, specifically the Kölliker-Fuse nucleus (KF) which is part of the parabrachial complex (PB) (Chatonnet et al., 2007); a neighboring landmark is the middle cerebellar peduncle (mcp) which courses through caudal r1 from ventral to dorsal (Franklin and Paxinos, 2008; Figures 1A,B, 2A, 3). Pr5 relates also rostrally with the ventral nucleus of the lateral lemniscus (VLL) within r1, separated from it by some fibers (Figure 2A).

The molecular mappings showed that the genes *Baiap3* and *Camk2a* are expressed selectively in KF, while they show no significant expression in Pr5 (Figures 4A,B,D and data not shown). On the other hand, *Irx2* is expressed by the Pr5 cells of r2, with no expression in KF (Figure 5A). These patterns thus delimit the r1/r2 boundary as the rostral end of the trigeminal column.

Another rostral component of the trigeminal system, the mesencephalic trigeminal nucleus can be observed across midbrain, isthmus and r1 in our AZIN2-lacZ sections (data not shown; see Martínez-de-la-Torre et al., 2018). We excluded this cell population from our analysis because it corresponds to primary sensory cells, therefore with a typology and category different from those of Pr5 and Sp5 (Hunter et al., 2001). An additional relation of the prepontine hindbrain region with the trigeminal system is represented by a small contribution to the motor trigeminal nucleus (5N) (Figures 1B, 3) as it can be deduced from the description in mid-gestational mouse embryos (Cordes, 2001).

### Pontine Region (r2, r3, and r4)

This region is primarily characterized by the mass of the migrated pontine nuclei aggregated at the ventral surface of r3 and r4; they originate at the rhombic lip from rhombomeres r6-r8 (Di Meglio et al., 2013; Tomás-Roca et al., 2016; Figure 3).

Dorsally these rhombomeres participate in the formation of the cochlear nuclei, with r2 and r3 contributing principally to the ventral component (VC) and r4 plus r5 principally to the dorsal cochlear nucleus (DC) (Farago et al., 2006). In our material, we could not discern these differential contributions nor the precise limits between these subnuclei, since they homogenously express AZIN2-lacZ (Figures 1A,B). The cochlear nuclear complex abuts rostrally directly the cerebellum at the r1/r2 limit, so that it was used as an additional landmark for that boundary in our analyzed material (Figures 1A, 2A, 4B, 5C).

The 5N motor nucleus is formed principally across r2 and r3 (Schneider-Maunoury et al., 1997; Song et al., 2006; Figures 1B, 4B, 5C), similarly to Pr5 as commented above. Interposed between these two nuclei there lies the interfascicular trigeminal nucleus (IF5), formerly known as the tensor tympani part of the motor trigeminal nucleus (Fu et al., 2013; Figure 5C and data not shown).

The trigeminal nerve root (5n) is located at r2 in 10.0 dpc mice (Cordes, 2001), but lies displaced from the center of the neuromere, next to the r2/r3 boundary. We believe that due to its massive progressive growth it possibly invades the rostral part of r3 at later stages, with fibers encapsulating the r3 portion of Pr5 (Figures 1A, 2A,C). In this respect, it is known that the trigeminal ganglion continues its development and neurogenesis through gestational and postnatal stages up to even the adult stage (Lagares et al., 2007; Lanier et al., 2009) involving thus a progressive increase of afferent fibers of its entry root that may lead to the extension of the latter into r3.

After entering the hindbrain, many trigeminal primary afferents bifurcate into ascending and descending branches (Cajal, 1909) which are assumed to reach, respectively, Pr5 and Sp5 cells (Aström, 1953; Usunoff et al., 1997; Waite, 2004). Considering the aforementioned known rhombomeric location of Pr5 in the mouse, it can be deduced that in this species the so-called ascending branches follow their path into Pr5 within r2 and r3 (Oury et al., 2006; Rhinn et al., 2013; Figures 2B,D) while the descending branches would extend caudalwards through the rest of rhombomeres, forming the spinal trigeminal tract.

The fibers of the facial nerve (7n) extend radially through alar r4, down to their exit point located ventrolaterally within this rhombomeric domain (Di Bonito et al., 2017; Martínez-de-la-Torre et al., 2018; Figure 1B). In r4 there is also the entry root of the vestibulocochlear nerve (8n) as observed in early embryos (Cordes, 2001); in the adult brain this root appears in a dorsolateral position slightly separated from the 7n root (Franklin and Paxinos, 2008; Di Bonito et al., 2017; Martínez-de-la-Torre et al., 2018; Figures 3, 5C, 6E). According to the aforementioned mapping of Pr5 to r2 and r3, the rostralmost portion of Sp5 lies within r4 (Figures 1A,B, 2A,C, 3).

Due to the ventrally convex pontine flexure of the hindbrain, the r2, r3 and r4 rhombomeres appear skewed and wedge-shaped, narrowing from ventral to dorsal, as seen in sagittal and parasagittal sections (Figures 1A, 2A,C, 3). Their dorsalmost portions correspond to their respective parts of the alar cochlear nuclei and the choroidal roof (not shown), which are pushed backward by the large mass of the cerebellum. Their backward inclination causes that sections in the conventional coronal plane cut obliquely through these rhombomeres (e.g., Figures 4B, 5C,D, 6C–F).

As regards our molecular characterization, *Irx2* is expressed in the r2 portion of Pr5, with some disperse cells within r3, as can be observed in adult and juvenile sections (Figures 5A–H and Table 1). Therefore, this gene marks a rhombomeric subdivision of Pr5. Additionally, in some sections *Baiap3* appears expressed in scarce cells in r2 as compared to r3 (data not shown).

In the adult *Kcng4* is expressed in the r2 and r3 portions of the trigeminal column, that is, the territory corresponding to Pr5 (Figures 6A–D,G,H), showing additionally some disperse positive cells within Sp5 at r4 (Figures 6A,B,E,F). This pattern appeared also in juvenile stages, although with an apparently regionalized pattern within Pr5, so that the ventral portion of r2 lacked expression of this gene (Figures 6G,H). Besides their different density, the cells marked within r4 showed a larger size as compared to those in r3, at least as observed in adult parasagittal and coronal sections (Figures 6A–F).

Similarly, *Calb2* is significantly expressed in the r2 and r3 portions of Pr5, together with some positive cells in r4 (Figures 10J–L). *Calb2*, *Irx2* and *Kcng4* display also expression in more caudal hindbrain regions, as commented below.

### Retropontine Region (r5 and r6)

This region is characterized, among other features, by the abducens motor nucleus (6N) and the facial nerve genus in r5, and the facial ascending fibers (asc7) plus the migrated facial motor nucleus (7N) in r6 (Figures 1B, 3; Cordes, 2001; Tomás-Roca et al., 2016; Martínez-de-la-Torre et al., 2018). 7N bulges somewhat from r6 into the adjoining r5 and r7 segments, deforming the interrhombomeric boundaries rostrally and caudally (Figure 3) as deduced from the fate mapping based on Hox gene expression (Tomás-Roca et al., 2016). The rostral limit of r5 lies just caudal to the fiber bundles of 7n and 8n in r4, as observed in sagittal and coronal sections of adult brains with transgenic lineage tracings (Di Bonito et al., 2017; Watson et al., 2017).

The parts of Sp5 corresponding to rhombomeres r5 and r6 strongly express *Mafb* (Figures 7A–C,F,G) and *Fn1* (Figures 8A–D,G,H), with a higher intensity than the rest of rhombomeres. There appears moderate expression of both genes rostrally at r4 and caudally through r7 and r8, while the caudal rest of rhombomeric trigeminal portions contains sparse positive cells (Figures 7A–E,H,I, 8A–F,I,J). *Fn1* displays a negative zone in the dorsalmost part of Sp5 extending from r4 to r8, probably corresponding to the dorsomedial subdivision of the trigeminal spinal nucleus (DMSp5) (Figures 8G,H).

### Medulla Oblongata (r7–r11)

The cryptic segments within this region (lacking visible boundary constrictions) were first discovered through fate mapping in avian chimeras (Cambronero and Puelles, 2000). They were subsequently corroborated by analysis of step-like changes in Hox expression patterns (Marín et al., 2008; Tomás-Roca et al., 2016). The medullary region is characterized by longitudinal plurisegmental structures such as the inferior olive (IO), found ventrally from r8 to r11, the complex formed by the hypoglossal nucleus (12N) close to the midline, plus the migrated vagal preganglionic motor nucleus (10N) from r9 to r11, and the ambiguous branchiomotor nucleus (Amb) extending caudalwards to the 7N in a deep intermediate position from r7 to r10 (Figure 3). The medulla comprises as well sizeable portions of the trigeminal, viscerosensory (solitary), and vestibular columns in the alar plate, and raphe nuclei at or next to the ventral midline (Alonso et al., 2013). The caudalmost rhombomere, r11, abuts the first myelomere or spinal cord segment (my1) occupied ventromedially by the pyramidal decussation (pyx). The rostrocaudal axis of the medulla oblongata follows more or less a straight horizontal direction. However, its caudal end, together with the upper spinal cord, forms the cervical flexure. Its pronounced curvature (together with possible variations in the angle of the sectioning plane) causes that coronal sections through r11 or even r10 cross obliquely the hindbrain/spinal cord (r11/my1) boundary, including thus the pyx in the same sections than the caudalmost rhombomeres.

Sp5 extends from r4 to r11 as commented above (Figures 1A–C, 2A,C). Therefore, the descending trigeminal primary afferents, from their entry point in r2 (or probably r2 plus the upper r3, as commented above) extend along the whole length of this nucleus as the spinal trigeminal tract, which continues into the spinal dorsal column and marginal stratum of the dorsal horn at least along the first 2 spinal segments. The dorsal horn of this upper portion of the spinal cord is accordingly described as part of Sp5C, considering also that their laminated cytoarchitecture is almost identical (Usunoff et al., 1997; Ten Donkelaar, 2020). The trigeminal afferents coming from the original branches of the trigeminal nerve are ordered dorsoventrally in the ascending and descending trigeminal tracts, so that dorsally there appear the mandibular fibers (sp5md) and ventrally the ophthalmic fibers (sp5ot) (Figures 1A,B, 2A,C) with the maxillary fibers in an intermediate position (data not shown) (Waite, 2004). These three bundles of fibers remarkably display different expression levels of the AZIN2-lacZ transgene (compare spt5md and sp5ot in Figure 1A) as previously described in coronal brain sections of these mice (Martínez-de-la-Torre et al., 2018).

Concerning the classical regionalization of Sp5, the boundary between the interpolar and the caudal subnuclei apparently lies at the r9/r10 transition, as was deduced from the Hox-based mapping of rhombomeres in mice (Tomás-Roca et al., 2016). This boundary is easily recognizable histologically attending either to their cytoarchitecture or to their pattern of afferent fiber terminals. A clearcut transition occurs from the non-laminar interpolar Sp5 to the laminar caudal Sp5 (Figures 2A,C; Olszewski, 1950; Waite, 2004). On the other hand, the boundary between the interpolar and oral subnuclei of Sp5 is uncertain on a morphological basis, so that in the literature it has been located tentatively at different positions (see section “Discussion”).

The dorsal horn of the first 2 myelomeres or cervical segments (C1 and C2), is functionally a continuation of the caudal subnucleus of Sp5, as commented above; it receives the terminals of the descending primary trigeminal afferents, and has a largely similar cyto- and myeloarchitectural aspect. In our molecular mappings we noted a continuous pattern from r10/r11 to my1 concerning the expressed genes and their laminar pattern, so that in our figures showing gene expression we have drawn the contour of Sp5 as including the dorsal horn of my1. The eventual continuation of this pattern into my2 could not be ascertained in the studied material, since seemingly the analyzed sagittal or coronal brain sections included only down to my1.

Among the genes we mapped, *Baiap3* (Figures 4A–C) and *Tac1* (Figures 9A–C) display specific expression in the region formed by r10, r11, and my1, corresponding to the caudal subnucleus of Sp5 as commented above. This labeled region limits rostrally with r9 that remains negative, corresponding to the caudal end of the interpolar Sp5. Both genes appear expressed in the superficial zone of the caudal Sp5 subnucleus, corresponding to the marginal and gelatinous layers (laminae I and II), as previously reported for substance P (product of the *Tac1* gene) (Del Fiacco and Cuello, 1980; Ribeiro-da-Silva and Hökfelt, 2000). There appear also disperse *Tac1* positive cells from r2 to r9 within the rest of the trigeminal column (Figures 9A–C and data not shown). *Baiap3* and *Tac1*, as well as other genes commented below, display an uniform pattern along the rostrocaudal axis of the region formed by r10, r11 and my1, so that they cannot be used as markers for the delimitation of the r10/r11 and r11/my1 boundaries. Therefore, in the image series showing their expression we have traced these intersegmental boundaries tentatively, following the results of Hox gene expression (Tomás-Roca et al., 2016; see section “Discussion”).

*Tac2* is expressed only in the gelatinous layer of the caudal subnucleus of Sp5 (Figures 9D–F). It displays a regionalized pattern within this subnucleus, with substantially fewer positive cells in r10 than in r11 or my1, as observed across juvenile and adult stages (Figures 9D,F; also compare Figure 9E with Figure 9B, respectively, showing *Tac2* and *Tac1* in r10). Therefore, from the analyzed genes *Tac2* is the only one displaying a rostrocaudal regionalized pattern within the caudal Sp5, as compared with the homogeneous rostrocaudal expression of the aforementioned *Baiap3* and *Tac1*, as well as *Calb1*, *Calb2*, and *Camk2a* as commented below.

*Calb2* signal also appears within Sp5 (the r10 and r11 parts are positive, while r9 is negative) in the P14, P28, and adult brains. This marker is also expressed at these stages in discrete neuronal populations in r2 and r3, and in a less intense manner in disperse cells from r4 to r8 (Figures 10J–R and data not shown). In its turn, *Calb1* is expressed from r9 to r11 across all stages (Figures 10A–I). Although *Calb1* labels these three rhombomeres crossing the interpolar-caudal limit (r9/r10), the labeling in r9 is nuclear and homogeneous, whereas the expression pattern is laminar in r10 and r11, thus displaying the interrhombomeric limit (Figures 10A–C). *Camk2a* displays a similar pattern concerning r9, r10 and r11 (Figures 4D–G). On the other hand, a vertical palisade of *Calb1*-negative cells was distinguished along the aforementioned r9/r10 limit, enhancing its visibility and pointing to a specific cell population at this precise location (Figures 10A–C and Supplementary Figure 1).

The r9 portion of Sp5 appears labeled specifically by the expression of *Pde1c* (Figures 11A–D) and *Zbtb16* (Figures 11E–N) genes, in which cases the rest of the trigeminal column remains negative. This rhombomeric portion is also differentially labeled by the expression of *Camk2a* and *Calb1*, which show a homogenous moderate expression in contrast with the scattered cells at r8 and the laminar pattern of r10 and r11 (Figures 4D–G, 10A–I). The r9 part of Sp5 is also characterized by the absence of expression of *Calb2* as compared with adjacent rhombomeres (Figures 10J–R). Moreover, this portion of Sp5 expresses homogenously *Kcng4* in contrast to the scattered positive cells seen in r8 and the absence of expression in r10 and r11 (Figures 6A,B,G,H).

In our screen, we did not identify any specific molecular markers for the rostral rhombomeres of the medulla oblongata, r7 and r8. However, the r8 portion of Sp5 can be distinguished by a pattern of scattered intensely labeled *Calb1*-positive cells, which differentiates this segmental module from the pattern in r7 (with much less density and intensity of the labeling) and r9 (with a more homogenous expression than r8) (Figures 10A–G). In its turn, *Calb2* showed an heterogenous pattern in the rostral medulla (r7 and r8) that also extended into r4, r5 and r6, but did not allow to distinguish a rhombomeric periodic pattern after examination of the image series (Figures 10J–L and data not shown).

Considering the set of r7, r8 and r9 Sp5 domains (that is, the medullary part of Sp5, after excluding the caudal subnucleus), it is jointly characterized by *Irx2* expression, present as a shared pattern of scattered positive cells, in contrast with the absence of expression in the rest of Sp5 (the retropontine region -r5 and r6- and the caudal subnucleus -r10 and r11) (Figures 5E–H).

## Discussion

We have described genoarchitecturally a segmental pattern of the trigeminal column, including both Pr5 and Sp5, attending to morphologic landmarks of the different rhombomeres, and to the regionalized expression of genes that show specificity for one or several rhombomeric domains. Next, we comment our results in the context of previous studies and knowledge about trigeminal subdivisions, hindbrain segmentation and molecular markers of trigeminal neurons.

### Classical vs. Rhombomeric Subdivisions of the Spinal Trigeminal Nucleus

The subdivision of Sp5 into oral, interpolar and caudal subnuclei was first described in primates on a cytoarchitectural basis, according to cell size and morphology, as well as the density of the neuronal populations (Olszewski, 1950). This subdivision was extrapolated to other mammal species as well as sauropsides (Molenaar, 1978; Arends and Zeigler, 1989) and established as the basis for anatomical, pharmacological or physiological studies of this structure. Differences between these Sp5 subnuclei concerning expression of molecular markers, patterns of incoming trigeminal primary afferents, or connectivity with other brain regions have been reported (Waite, 2004).

Some of the boundaries of the classic Sp5 subdivisions can be identified with acceptable precision, and correlated with the rhombomeric map. The oral subnucleus abuts rostrally Pr5, which lies within r2 and r3 (Oury et al., 2006). The limit between Pr5 and the oral Sp5 subnucleus accordingly coincides with the r3/r4 limit. The caudal Sp5 subnucleus is continuous with the dorsal horn of the spinal cord, mapping thus down to the r11/my1 limit and extending into the upper two spinal cord segments (Usunoff et al., 1997; Ten Donkelaar, 2020). Other authors use a nomenclature that differentiates, as two components of the sensory trigeminal column, the caudal subnucleus (within the medulla oblongata) and the upper cervical dorsal horn, continuous at the hindbrain/spinal cord junction (Jacquin et al., 1986; Noma et al., 2008; Faunes and Wild, 2017). In its turn, the abrupt structural transition between the non-laminar interpolar Sp5 portion and the laminar caudal subnucleus allows the limit between them to be defined, which corresponds to the r9/r10 limit as identified by the Hox gene pattern (Tomás-Roca et al., 2016).

However, a problem arises when we try to differentiate between the oral and interpolar subnuclei of Sp5. They display subtle cytoarchitectural differences, but these apparently do not render a clearcut boundary, so that the precise morphological location of the oral/interpolar limit varies across the literature (Phelan and Falls, 1989). Besides, there are also differences between these two subnuclei concerning molecular markers or connectivity (Erzurumlu and Killackey, 1979; Veinante et al., 2000; Waite, 2004; Ashwell et al., 2006). None of these studies showed a distinct boundary between them. In rodents it is conventionally assumed that the oral/interpolar limit lies at the level of the caudal pole of the facial motor nucleus (Phelan and Falls, 1989; Waite, 2004) so that following the rhombomeric map it would correspond to the r6/r7 limit. Accordingly, the oral and interpolar Sp5 subnuclei would consist, respectively, of the r4-r6 and r7-r9 portions of the plurineuromeric trigeminal column.

Nevertheless, the classic subdivision into oral, interpolar and caudal parts turned out to be insufficient to interpret the rostrocaudal organization of Sp5 in several studies. In his own seminal work proposing this subdivision, the oral subnucleus is described as containing a rostral subdivision with different cytoarchitecture (Olszewski, 1950). A detailed cyto- and myeloarchitectural study of the interpolar subnucleus shows significant differences between successive transversal sections of this structure (Phelan and Falls, 1989), so that these rostrocaudal differences may be explained by an internal segmental organization within this subnucleus. In birds the analysis of the pattern of trigeminocerebellar connections needed the further subdivision of the oral and interpolar subnuclei into two rostrocaudal subunits each (Arends and Zeigler, 1989). In the cat, the whole trigeminal column (Pr5 plus Sp5) was subdivided into 12 rostrocaudal units according to the peripheral origin of their primary trigeminal afferents (Marfurt, 1981). Interestingly the caudal subnucleus -including the upper cervical dorsal horn- can be subdivided into four rostrocaudal regions innervating, respectively, oral/perioral, snout, periocular, and periauricular receptive fields (Panneton et al., 2017). Studies about the central processing of pain have introduced an additional trigeminal rostrocaudal subdivision, identified as the interpolar/caudal transition zone, which specifically receives nociceptive afferents (Ren and Dubner, 2011).

Therefore, considering these multiple rostrocaudal subdivisions of Sp5, plus the modern concept of a neuromeric hindbrain (Tomás-Roca et al., 2016; Martínez-de-la-Torre et al., 2018), a rhombomere-based framework needs to be developed that should facilitate the finding and characterization of trigeminal functional units, which apparently remain partly hidden if only the tripartite classic schema is assumed. An advantage of the rhombomeric model is that it would be underpinned by modern embryological and molecular criteria. Such a model would be also a basis for interspecies comparison, taking into account the conservation of the rhombomeric (neuromeric) pattern across vertebrates.

### The Segmentation of the Trigeminal Column in the Medulla Oblongata

From the different proneuromeric regions of the hindbrain (prepontine, pontine and retropontine regions, and medulla oblongata; Puelles et al., 2013, 2018) the latter appears as the more extense, including as well more rhombomeres. The medulla oblongata has the peculiarity of lacking the typical interrhombomeric limits of the more rostral hindbrain. Such limits are visible as constrictions at early stages, and consist of specific neuroepithelial cell populations that act as barriers to clonal cell dispersion, and as a source of morphogens for adjacent rhombomeres, among other cellular and molecular characteristics (Pujades, 2020). Albeit the absence of these overt limits in the medulla oblongata, five rhombomeres (r7-r11) were proposed in this region, separated by cryptic limits antimeric to neighboring intersomitic boundaries, through experiments with quail-chick chimeras (Cambronero and Puelles, 2000). Ulterior analysis showed that r7-r11 are differentially characterized by the nested expression of Hox genes of the paralog groups 4–8, very much similarly as Hox genes of the paralog groups 1–3 delimit the overt rhombomeres r1-r6 (Marín et al., 2008; Tomás-Roca et al., 2016). Initially these 5 medullary rhombomeres were identified as “pseudorhombomeres” (Cambronero and Puelles, 2000), but later the descriptor “cryptorhombomeres” was thought to be more appropriate, since these units are not false neuromeres, but only hidden ones, only separated by given molecular properties.

Concerning the trigeminal column, the medulla oblongata contains the most conspicuous internal limit of this structure, which separates the interpolar and caudal subnuclei at the r9/r10 limit (Tomás-Roca et al., 2016; Martínez-de-la-Torre et al., 2018; Puelles et al., 2018). Morphologically this limit is visible as an interface between the homogenous nuclear cytoarchitecture of the interpolar subnucleus and the laminar pattern of the caudal subnucleus (Olszewski, 1950). This change correlates with the expression pattern of diverse genes (present data) including *Hoxa6* and *-b6* whose domains end rostrally at this limit, as we observed at perinatal stages (Tomás-Roca et al., 2016). Physiologically, this limit is highly relevant considering the involvement of the caudal subnucleus in the central processing of craniofacial pain (Bereiter et al., 2000).

The caudal subnucleus (Sp5C) shows apparently an uniform cyto- and genoarchitecture through its r10 and r11 portions, which also extend with little variation into my1, according to previous literature (Olszewski, 1950; Bereiter et al., 2000; Waite, 2004) now corroborated by our data on gene expression. Nevertheless, we observed that the expression of *Tac2* shows a gradient-like pattern with decreasing positive cells from my1 into r10. At perinatal stages the r11 part of the caudal subnucleus is positive for *Hoxb7* while the r10 part is negative. The domain with highest expression of *Hoxb8* reaches up to the r11/my1 limit, with additional weaker expression within r11 (Tomás-Roca et al., 2016). Taken jointly, these data suggest the existence of subtle molecular differences starting with differential Hox gene properties within the medullary and spinal portions of Sp5C, which may lead to functional properties that would need to be explored.

Within the medulla oblongata, the rhombomere that was singled out more directly by our approach is r9, considering its specific expression of *Ped1c* and *Zbtb16*, as well as its distinctive pattern in relation to *Calb1*, *Calb2, Camk2a*, and *Kcng4* expression, as commented in Results. This rhombomere represents the rostralmost expression domain of paralog *Hox5* genes (Tomás-Roca et al., 2016). It may correspond to the part of the interpolar subnucleus that is reactive to orofacial nociceptive stimuli defined as the so-called interpolar/caudal transition zone (Ren and Dubner, 2011).

It would remain to be assessed the possible individuality at molecular level of the r7 and r8 portions of Sp5, which are quite similar structurally and molecularly, as commented in Results. Another case of a pair of rhombomeric domains with relative molecular similarity is that of r5 and r6, which share specific *Mafb* and *Fn1* expression. However, the r6 portion of Sp5 has a specific trigeminocerebellar projection in the chick (Díaz and Puelles, 2003) so that further research could yield molecular and functional differences between r5 and r6, as well as between r7 and r8. On the other hand, at perinatal stages these pairs of rhombomeres are internally differentiated by Hox expression, with *Hoxd3* and Hox4 paralogs expressed up to the r5/r6 and r7/r8 limits, respectively (Tomás-Roca et al., 2016).

Therefore the medullary portion (r7-r11) of the trigeminal column consists of successive rostrocaudal units that largely fit the rhombomeric map, an organization also observed in the vagal motor nucleus (Cambronero and Puelles, 2000; Marín et al., 2008), the inferior olive (Hidalgo-Sánchez et al., 2012), the reticular formation (Gray, 2013) or the raphe nuclei (Alonso et al., 2013) all of them longitudinal structures that span several rhombomeres of the medulla oblongata. Its apparent early morphologic homogeneity or uniformity contrasts with the overt segmentation of the rostral half of the hindbrain (Parker and Krumlauf, 2020; Pujades, 2020; Krumlauf and Wilkinson, 2021). However, according to current evidence the medulla oblongata emerges as a complex region formed by multiple neuromeric units with differential molecular profiles (Nieuwenhuys and Puelles, 2016; Puelles et al., 2018; Watson et al., 2019).

### Molecular Markers With Regionalized Expression in the Trigeminal Column

Some of the molecular markers we report (*Calb1*, *Calb2*, *Camk2a*, *Tac1*, *Tac2*) have been previously described in relation to the regionalization of the trigeminal column, while others (*Irx2, Mafb, Fn1, Pde1c, Zbtb16, Kcng4*, and *Baiap3*) are novel in this respect.

In the rat, *Calb1* and *Calb2*, which code, respectively, for the calcium binding proteins calbindin and calretinin, are expressed in the caudal subnucleus, principally in its gelatinous layer or lamina II. Both are also expressed in disperse cells in the rest of the trigeminal column (that is, the Pr5, and the oral and interpolar Sp5 subnuclei) although the description of this pattern varies across the literature, probably due to different sensitivity or specificity of antibodies (Arai et al., 1991; Bennett-Clarke et al., 1992; Rogers and Résibois, 1992; Ashwell et al., 2006). A significant novelty of our study is the identification of a segment-like portion of the interpolar subnucleus (r9) that is positive for *Calb1* and negative for *Calb2*. This had not been noticed previously possibly because the scarce use of parasagittal sections in classical neuromorphological analyses.

Another calcium-related gene, *Camk2a* (calcium/calmodulin-dependent kinase II α) is functionally involved in nociceptive pathways, as deduced from the study of mutant mice (Zeitz et al., 2004). It was described as expressed in neurons of the marginal and gelatinous layers of the spinal dorsal horn, as well as in unmyelinated neurons of the trigeminal ganglion. We show now that its expression extends to the superficial layers of the caudal Sp5 subnucleus in r10 and r11, with a pattern similar to that described in the spinal cord, in addition to the expression in the non-laminar segment-like portion of the interpolar Sp5 in r9. This latter expression would support the correspondence of the r9 portion with the “caudal/interpolar transition zone,” which is involved in pain processing, as commented above.

*Tac1* and *Tac2* encode, respectively, the neurotransmitters substance P and neurokinin B. Both genes, or their protein products, have been described in the superficial laminae of the dorsal horn and caudal trigeminal subnucleus (Del Fiacco and Cuello, 1980; Ribeiro-da-Silva and Hökfelt, 2000; Mar et al., 2012). We have found that their expression patterns display segment-related differences, with *Tac1* reaching up to the r10 trigeminal portion in a homogenous pattern, and *Tac2* showing less positive cells in r10, as reported in Results.

*Irx2*, a member of the Iroquois family of transcription factors, has expression along the hindbrain in 10 dpc mouse embryos (Bosse et al., 1997; Cohen et al., 2000) so that the pattern we describe in the trigeminal column would be derived from that early expression. Interestingly *Irx2* differentiates the r2 from the r3 portion of Pr5, as well as a medullary portion of Sp5 (r7-r9) that would correspond to the interpolar subnucleus as described in the classical nomenclature. Given the known role of the Iroquois gene family in pattern formation and differentiation (Gómez-Skarmeta and Modolell, 2002) it would be expected the involvement of Irx2 in trigeminal development. It would exist also the possibility of a role in the differentiated mature neurons where it is expressed.

*Mafb* is selectively expressed in r5 and r6 of the early developing hindbrain as well as in several brainstem structures such as a part of the cochlear nuclei (Cordes and Barsh, 1994; Eichmann et al., 1997). *Mafb* is necessary for the normal development of these rhombomeres through the regulation of the developmental genes *Krox20* and *Fgf3*, and several *Hox* genes (Frohman et al., 1993; Manzanares et al., 1999, 2002; Giudicelli et al., 2003), all of which are involved in hindbrain segmentation. Our results show that *Mafb* maintains its expression in the trigeminal derivatives within r5 and r6 in the adult brain, suggesting additional roles in these neuronal populations.

*Fn1* encodes the extracellular matrix component fibronectin. Its mRNA expression appears in telencephalic migrating neurons while in the adult brain it is restricted to the subiculum (Sheppard et al., 1995; Kashima et al., 2019). Here, we describe that it is additionally expressed at least within given segmental portions of the trigeminal column. Its possible function in these neuronal populations would need to be tested.

*Pde1c* encodes the calmodulin-dependent cyclic nucleotide phosphodiesterase and is expressed in olfactory sensory neurons (Yan et al., 1995), in migrating neurons in the cerebellum and cerebral cortex (Gong et al., 2003), and in lamina I neurons of the dorsal horn involved in nociception, principally at lumbar levels (Torsney et al., 2006). Interestingly, our results show a specific expression of this gene in the r9 part of Sp5, delimiting the caudal/interpolar transition zone, involved in pain signaling.

*Zbtb16*, also known as promyelocyte leukemia zinc finger (PLZF), was identified in an unique case of acute promyelocytic leukemia (APL), where it is fused to the retinoic acid receptor cx (RARor) (Chen et al., 1993). *Zbtb16* is characterized by a dynamic pattern of expression across multiple regions of the neural tube (Avantaggiato et al., 1995; Cook et al., 1995). PLZF is a transcriptional regulator of *Hox* genes during hindbrain development (Ivins et al., 2003). We have described a novel feature of the expression pattern of this gene, namely its specific location in a single rhombomeric domain (r9), similarly to *Pde1c*.

*Kcng4* encodes a subunit of a potassium voltage-gated channel that has been identified as potentially linked to migraine (Lafrenière and Rouleau, 2012). Although this brain disorder has been extensively linked to the trigeminal system, and mainly to the caudal Sp5 subnucleus (Ashina et al., 2019; Messlinger and Russo, 2019), the pathophysiology of chronic migraine is not fully understood. Our work shows that the migraine-related gene *Kcng4* is actually expressed from r2 to r9, which correspond to the principal, oral and interpolar subnuclei, which also have recently been proposed to play a role in migraine (Youn, 2018).

*Baiap3* is a member of the mammalian uncoordinated 13 (Munc13) protein family of synaptic regulators of neurotransmitter exocytosis (Shiratsuchi et al., 1998; Koch et al., 2000). In the human brain, its mRNA is expressed in several regions including the cortex, amygdala, hypothalamus and periaqueductal gray (Shiratsuchi et al., 1998; Lauridsen et al., 2011; Wojcik et al., 2013). Knockout mice and given polymorphisms for this gene lead to or are correlated with anxiety behavior and substance abuse (Wojcik et al., 2013). We have described a strong and specific expression in the caudal Sp5 trigeminal nucleus, so that it might have a role in the nociceptive function of this region.

## Conclusion, Limitations, and Future Research

As commented above, we have tentatively traced the interrhombomeric boundaries within the mouse trigeminal sensory column according to current knowledge collected in the prosomeric model, based either on descriptive morphologic or molecular gene expression data, or on experimental approaches involving fate maps with avian chimeras or transgenic mice. Some of the dispersed sparse populations described in some parts of the trigeminal column may correspond to cells migrated tangentially from neighboring rhombomeric modules where a similar labeling is massive. Obviously, in order to ascertain precisely our delimitation of the rhombomeric domains, as well as possible cell migration or intermingling across rhombomeric limits, it would be necessary to perform future *ad hoc* experiments. These would ideally combine our reported genetic markers of trigeminal subdivisions with experimental fate maps for each rhombomere.

One difficulty we encountered was the obliquity of some interrhombomeric limits due to the pontine and cervical flexures of the neural tube (Figure 1A). Moreover, it should be remembered that the rhombomeric domains are known to adopt a degree of obliquity with respect to the mediolateral axis (Figure 1B) with their medial parts displaced anteriorly with respect to their lateral portions. These circumstances cause that standard coronal sections are necessarily variously oblique in relation to rhombomeric domains and their limits. We have tried to solve this handicap in coronal sections by taking into account the well-known rhombomeric landmarks found relatively close to the trigeminal column, like the trigeminal and facial motor nuclei, or the fibers of the facial and vestibulocochlear nerves. Additionally, our rhombomeric map is consistent with the observations in sagittal sections, which display well some of the mentioned deformations.

An issue that would require further insight is the possible dorsoventral regionalization within each of the trigeminal rhombomeric domains related to mandibular, maxillary and ophthalmic inputs (e.g., the dorsomedial portion of the Pr5 and Sp5), together with a more exhaustive molecular characterization of the radial layering in the caudal subnucleus of Sp5. It would also deserve further study the transition from the medullary to the cervical portions of the caudal subnucleus, considering that it crosses over such a significant landmark as the brain/spinal cord junction.

Overall, we have succeeded in reporting segmental (transverse, rostrocaudally ordered) subregions along the trigeminal column based on molecular and fate mapping criteria delimiting rhombomeric domains. Our results are reliable since they are reproduced from sagittal to coronal section series of the adult brain, together with data on juvenile stages. We focused this analysis on the adult brain, so that the segmental map should not be regarded as a transient developmental state but as an instance of the neuromeric pattern persisting in the mature brain. This mapping of the trigeminal system can provide the basis for further functional and/or pharmacological studies considering the molecular and embryological uniqueness of the different rhombomeric subdivisions of this structure.

## Data Availability Statement

Publicly available datasets were analyzed in this study. This data can be found in: The Gene Expression Nervous System Atlas (GENSAT) Project, NINDS Contracts N01NS02331 and HHSN271200723701C to The Rockefeller University (New York, NY), available from www.gensat.org; © 2004 Allen Institute for Brain Science, Allen Mouse Brain Atlas, available from mouse.brain-map.org, and ©2008 Allen Institute for Brain Science, Allen Developing Mouse Brain Atlas, available from developingmouse.brain-map.org.

## Ethics Statement

The animal study was reviewed and approved by the University of Murcia Committee for Animal Experimental Ethics.

## Author Contributions

FM conceived the research. IG-G and FM performed the data mining. MM-D-L-T prepared and processed the brain sections from AZIN2-lacZ mice. IG-G, PA, and FM analyzed the data and performed the image analysis and figure preparation. IG-G, PA, LP, and FM wrote the manuscript. All authors contributed to the article and approved the final submitted version.

## Conflict of Interest

The authors declare that the research was conducted in the absence of any commercial or financial relationships that could be construed as a potential conflict of interest.

## Publisher’s Note

All claims expressed in this article are solely those of the authors and do not necessarily represent those of their affiliated organizations, or those of the publisher, the editors and the reviewers. Any product that may be evaluated in this article, or claim that may be made by its manufacturer, is not guaranteed or endorsed by the publisher.

## Footnotes

1
mouse.brain-map.org

2
developingmouse.brain-map.org

## Abbreviations

5N: trigeminal motor nucleus
5n: trigeminal nerve root
5Sol: trigeminal-solitary transition zone
6N: abducens motor nucleus
7N: facial motor nucleus
7n: facial nerve
8n: vestibulocochlear nerve
10N: vagal motor nucleus
12N: hypoglossal motor nucleus
Amb: ambiguous branchiomotor nucleus
AP: area postrema
asc7: facial ascending fibers
CBN: cerebellar nuclei
CBX: cerebellar cortex
DC: dorsal cochlear nuclei
DMSp5: dorsomedial subdivision of the trigeminal spinal nucleus
ECu: external cuneatus nucleus
IF5: interfascicular trigeminal nucleus
IO: inferior olive
KF: Kölliker-Fuse nucleus
LRt: lateral reticular nucleus
mcp: middle cerebellar peduncle
Mx: matrix region of the medulla
my1: myelomere 1
PB: parabrachial complex
Pn: pontine nuclei
Pr5: principal trigeminal sensory nucleus
pyx: pyramidal decussation
r: rhombomere/s
Rt: reticular formation
SC: spinal cord
SO: superior olive
Sol: solitary nucleus
Sp5: spinal trigeminal sensory nucleus
Sp5C: subnucleus caudalis of the spinal trigeminal sensory nucleus
Sp5I: subnucleus interpolaris of the spinal trigeminal sensory nucleus
sp5md: mandibular fibers of the trigeminal tract
Sp5O: subnucleus oralis of the spinal trigeminal sensory nucleus
sp5ot: ophthalmic fibers of the trigeminal tract
SpVe: spinal vestibular nuclei
tz: trapezoid body
VC: ventral cochlear nuclei
Ve: vestibular nuclei
VLL: ventral nucleus of the lateral lemniscus.
