# Supplementary figures and images for: Molecular Segmentation of the Spinal Trigeminal Nucleus in the Adult Mouse Brain
Source: Front Neuroanat. 2021 Dec 10;15:785840. doi: 10.3389/fnana.2021.785840 (PMC8702626; doi:10.3389/fnana.2021.785840)

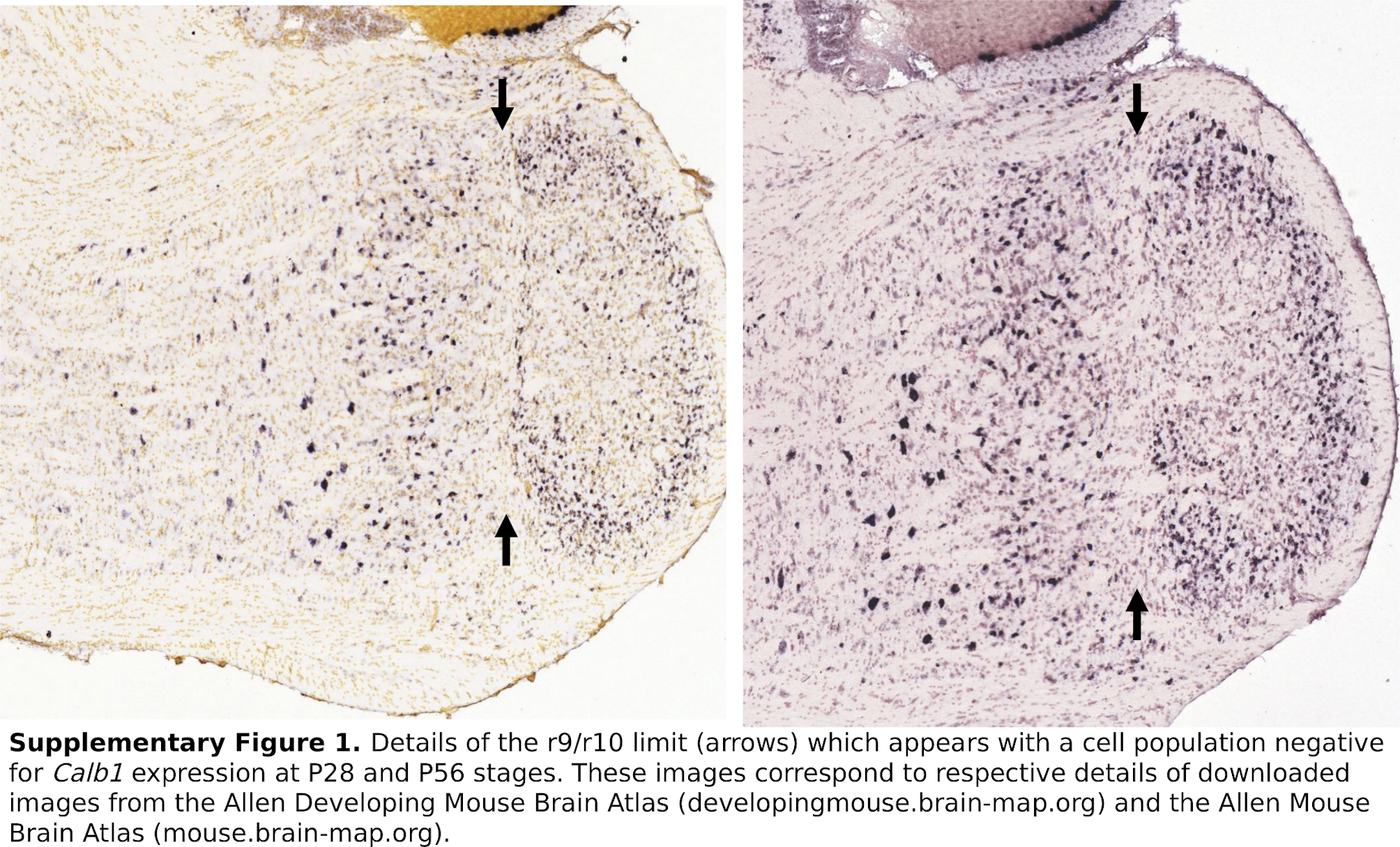

Supplement: Supplementary file 1 [file Image_1.tiff]
